# First‐in‐human safety, tolerability, pharmacokinetics and pilot food‐effect study of the candidate antimalarial compound MMV367

**DOI:** 10.1002/bcp.70000

**Published:** 2025-02-16

**Authors:** Andrea Kuemmerle, Nand Singh, Denis Gossen, Annick Janin, Raman Sharma, Anthony Cahn, Rachel A. Gibson, Somasekhara R. Menakuru, Erin Lambourne, Tom Dove, Francisco‐Javier Gamo, Laura Sanz, Benoit Bestgen, Stephan Chalon

**Affiliations:** ^1^ MMV Medicines for Malaria Venture Geneva Switzerland; ^2^ Quotient Sciences Nottingham UK; ^3^ Mangareva SRL, Kraainem Belgium; ^4^ AKJ Consulting, Divonne France; ^5^ GSK, Clinical Pharmacology and Simulation, R&D Stevenage UK; ^6^ GSK, Global Health Medicines, R&D Stevenage UK; ^7^ GSK, Global Health Medicines, R&D Tres Cantos Spain

**Keywords:** first‐in‐human, GSK3772701, malaria, MMV367, pharmacokinetics, safety, tolerability

## Abstract

**Aim:**

To evaluate the safety, tolerability and pharmacokinetics in healthy participants of orally administered MMV367 (GSK3772701), a novel antimalarial interfering with *Plasmodium falciparum* acyl coenzyme A synthetase 10/11 function.

**Methods:**

This first‐in‐human study enrolled 47 healthy male and female participants. Part 1 was a randomised, double‐blind, placebo‐controlled study in which four sequential fasted cohorts received MMV367 single ascending doses (100, 300, 750 and 1500 mg) or placebo (six active, two placebo per cohort). Part 2 was a randomised, open‐label crossover (fed‐fasted) pilot food‐effect study of MMV367 440 mg (n = 8). In Part 3 MMV367 400 mg was administered once daily for 3 days in a single cohort (six active, two placebo).

**Results:**

Treatment‐emergent adverse events (TEAEs) occurred in 36.8% (14/38) of participants receiving MMV367 *vs* 44.4% (4/9) with placebo. There were two MMV367‐related TEAEs, and no serious or severe TEAEs or clinically relevant changes in electrocardiograms, vital signs or laboratory tests. In Part 1 (fasted), maximum plasma concentrations occurred between 2.0 and 5.0 h post dose, with a geometric mean half‐life of 16.5‐18.4 h. Approximate dose proportionality was demonstrated across the dose range (100‐1500 mg). In Part 2, MMV367 relative bioavailability (fed *vs* fasted) was 161.4% (90% confidence interval 148.3, 175.6) for maximum observed concentration (*C*_max_), 130.4% (122.2, 139.1) for the area under the curve (AUC) until the last measurable concentration and 132.9% (124.1, 142.3) for AUC extrapolated to infinity. In Part 3, geometric mean day 1:3 exposure ratios (geometric co‐efficient of variability) were 1.9 (4.9%) for *C*_max_ and 2.1 (7.7%) for the AUC for the defined interval between doses after once‐daily dosing for 3 days.

**Conclusions:**

MMV367 demonstrated acceptable safety, tolerability and pharmacokinetic profiles supporting further development as an antimalarial drug.

**What is already known about this subject**

- Malaria is a protozoan disease with 3.4 billion people at risk globally. *Plasmodium falciparum* accounts for around 97% of all malaria cases and deaths.
- Pyrrolidinamides are a novel class of antimalarial drug targeting parasite acyl coenzyme A synthetase 10/11. Conditional knockdown experiments showed parasite acyl‐CoA synthetase 10 to be essential in asexual blood stage parasites.
- MMV367 (also named GSK3772701, GSK701 or MMV1582367) is a pyrrolidinamide antimalarial candidate demonstrating rapid activity against *P. falciparum*, no cross‐resistance to current antimalarial drugs and predicted to have appropriate oral availability.

**What this study adds**

- MMV367 had an acceptable safety profile and was well tolerated.
- Pharmacokinetics indicate an elimination half‐life of 15.9‐19.7 h, a 1.3‐1.6‐fold increase in exposure after a high‐fat meal and approximately 2‐fold greater exposure on day 3 compared to day 1 after once‐daily dosing for 3 consecutive days.
- MMV367 has potential for the treatment of uncomplicated *P. falciparum* malaria.

## 1 INTRODUCTION

Malaria threatens over 3.4 billion people, causing an estimated 263 million cases and 861 000 deaths in 2023, mainly caused by *Plasmodium falciparum*.^1^ Children under 5 years old and pregnant women, are particularly at risk.^1^, ^2^ Prompt diagnosis and treatment with artemisinin‐based combination therapies are key to case management, but emerging resistance to artemisinin and partner drugs in Africa represents a significant threat.^1^ Developing new antimalarial treatments combining drugs with novel mechanisms of action is essential to address this challenge.

Pyrrolidinamides are a novel antimalarial class identified by high‐throughput phenotypic screening of GSK's compound library at Tres Cantos.^3^ **Plasmodium falciparum acyl‐CoA synthetase** as designated by International Union of Pharmacology (IUPHAR) has been identified as a key target for antimalarial drug development.^4^ Pyrrolidinamide mode of action studies revealed that mutations in acyl‐CoA synthetase 10/11 modulate their potency, and that acyl‐CoA synthetase 10 is essential for asexual blood‐stage parasites, exposing a new parasite vulnerability.^3^ The pyrrolidinamide **MMV367** (also known as GSK3772701/GSK701/MMV1582367)^4^ demonstrated rapid activity against *P. falciparum* asexual blood stages, with no cross‐resistance to current antimalarial drugs in preclinical studies (data on file, GSK/MMV). Validated preclinical models demonstrated that MMV367 has oral bioavailability and fast parasite killing kinetics, indicating potential for the treatment of uncomplicated *P. falciparum* malaria (data on file, GSK/MMV).

This first‐in‐human study evaluated MMV367 safety, tolerability and pharmacokinetics (PKs) when administered orally to healthy adult participants as single ascending doses or multiple doses, including a pilot food‐effect study.

## 2 MATERIALS AND METHODS

### 2.1 Study drug

The investigational medicinal product (IMP), MMV367 dispersible granules (250 mg/g, 25% w/w), and matching placebo were manufactured by Piramal Pharma Solutions Ltd, Ahmedabad, India. Study medications were administered with up to 500 mL of water under observation.

### 2.2 Study oversight and ethics

The study complied with the International Council for Harmonization Good Clinical Practice Guidelines (E6R2), all applicable laws and regulations, and the Declaration of Helsinki. The protocol was approved by the Medicines and Healthcare products Regulatory Agency, UK, and the NHS Health Research Authority ethics committee. The clinical protocol is registered at **ClinicalTrials.gov** , identifier NCT05507970. The study was conducted between 8 July 2022 and 25 January 2023 at Quotient Sciences, Nottingham, UK. All participants provided written informed consent prior to study inclusion.

### 2.3 Clinical trial design

This first‐in‐human, phase 1 study in healthy participants was conducted in three parts.

Part 1 was a double‐blind, randomised, placebo‐controlled, single ascending dose study. Four sequential cohorts with eight participants in each cohort were randomised (3:1) to receive MMV367 (100, 300, 750 or 1500 mg) or placebo. In each cohort, a sentinel dosing strategy was used, with two participants (one MMV367, one placebo) observed for 96 h before the remaining participants in the cohort were dosed. Participants were admitted to the clinical unit the day before dosing. After an ≥8 h overnight fast, participants were dosed orally on day 1 with MMV367 or placebo and discharged on day 5, returning on days 7 and 15 (± 1 day) for follow‐up assessments. Following the completion of each dosing cohort, the safety advisory committee reviewed PK and safety data before progression to the next highest MMV367 dosing cohort. Part 1 also included a palatability assessment.

As per regulatory guidance, the MMV367 dosing rationale was selected based on the no‐observed‐adverse‐effect‐level (NOAEL) and associated exposures from 7‐day Good Laboratory Practice (GLP) studies in rats and dogs, as well as on predicted exposure in humans using allometric scaling and physiologically‐based PK (PBPK) modelling.^5^, ^6^ The starting dose in Part 1 (100 mg) was based on the NOAEL in the most sensitive species, female rats, with body weight loss and reduced food intake observed above 100 mg/kg/day. In dogs, the most sensitive species for drug exposure, vomiting, reductions in food intake and weight loss were observed above the 300 mg/kg/day NOAEL. With an estimated human bioavailability of 90%, safety margins were ~60‐fold for the area under the curve (AUC) and ~30‐fold for *C*_max_ compared to the dog NOAEL (data on file, GSK/MMV), with the highest dose limited to human exposures at least 3‐fold lower than the NOAEL in dogs. Dose selection in Parts 2 and 3 used PBPK modelling of human PK data from Part 1 and pharmacodynamic (PD) data from a validated preclinical malaria model (SCID mice with *P. falciparum*‐infected red blood cells), with an estimated MMV367 minimally efficacious human single dose of 440 mg (data on file, GSK/MMV).

Part 2 was an open‐label, randomised, balanced, two‐treatment, two‐period crossover pilot food‐effect study in eight healthy participants who received a single MMV367 440 mg dose on two occasions. This dose, established from Part 1, was predicted to have acceptable safety and tolerability, assuming a maximum 3‐fold exposure increase in the fed state. Participants were admitted the day before dosing, dosed on day 1 (period 1) and day 8 (period 2), discharged on day 12, with follow‐up on day 14 (± 1 day). In the first period, after an overnight fast (≥8 h), participants were randomised to receive MMV367 either fasted or after a high‐fat breakfast.^7^ Following a minimum 7‐day washout, they crossed over to the alternative condition.

Part 3 was a double‐blind, randomised, placebo‐controlled, multiple‐dose study. After an overnight fast, eight participants were randomised (3:1) to receive placebo or MMV367 400 mg once daily for 3 days, reflecting the duration of standard‐of‐care antimalarial therapy. The 1200‐mg total dose was based on PK modelling of Part 1 data, but was below the maximum dose tested in Part 1 (1500 mg). Sentinel dosing, as per Part 1, was used. Participants were dosed on days 1, 2 and 3, remained in the clinical unit until day 7, and returned on days 9 and 17 (± 1 day) for end‐of‐study assessments.

### 2.4 Participants

Participants were screened within 28 days of admission to the clinical unit on day −1. Eligible participants were healthy males or females (including those of childbearing potential), aged 18‐55 years, weight ≥50 kg and body mass index 18.0‐32.0 kg/m^2^. All participants had to use highly effective contraception.^8^ Exclusion criteria were any medical history, clinical or laboratory findings, or lifestyle factors that would increase participant risk or affect results interpretation, recent participation in a clinical research study, or concomitant medications, except hormonal contraception or hormonal replacement therapy.

### 2.5 Study objectives

The primary objective was to assess the safety and tolerability of MMV367 single and multiple oral doses in healthy participants. Secondary objectives were to assess MMV367 plasma PK following single and multiple doses, and the effect of a high‐fat meal on PK following a single MMV367 dose. Exploratory objectives were to assess the urine PKs of a single dose of MMV367, investigate MMV367 metabolite(s), and evaluate the taste attributes and overall acceptability of MMV367 dispersible granules. Additionally, although the GLP safety pharmacology studies in beagle dogs indicated no significant effect of MMV367 electrocardiogram (ECG) parameters at doses up to 1000 mg/kg (data on file, GSK/MMV), the MMV standard drug development pathway includes exposure‐response modelling for potential effects on QT interval (to be published separately).

### 2.6 Safety and tolerability assessments

The safety population included all participants who received MMV367 or placebo. Post‐baseline safety assessments across all three study parts included physical examinations and vital signs monitoring, safety laboratory investigations and urinalysis. Orthostatic vital signs were assessed during Parts 1 and 3. Single and triplicate ECGs were conducted in all study parts, with ECG telemetry monitored from 30 min post dose to 12 h post dose in Parts 1 and 2. In addition, to permit QT interval exposure‐PD analysis, Holter monitoring was conducted from 1.5 h post dose to 24 h post dose in all three parts.

Treatment‐emergent adverse events (TEAEs) were assessed throughout the study and categorised according to the Medical Dictionary for Regulatory Activities (MedDRA, version 25.1), with severity classified according to the study protocol. TEAE relationship to IMP/placebo was assessed by the investigator based on timing and clinical context. Clinically significant abnormalities in laboratory parameters, vital signs or ECGs were reported as adverse events. ECGs, Holter monitoring and telemetry were evaluated at a single centre (Banook, Nancy, France).

In Part 1, participants received training in taste test assessment and completed a written palatability questionnaire evaluating smell, sweetness, bitterness, flavour, mouthfeel/texture, grittiness and aftertaste on a 9‐point Likert scale completed individually, in private, immediately after dosing.^9^ Participants held the dosing solution in their mouths and swirled for 10‐15 s before swallowing.

### 2.7 PK sampling and analysis

In Parts 1 and 2, blood samples for plasma PK assessments were taken 0.5 h predose and at 14 time points until 144 h post dose. In Part 3, samples were collected 0.5 h before each dose, at nine time points following the first dose and three time points after the second dose until 12 h post dose, and at 14 time points following the third dose until 144 h post dose. Urine collections for PK assessment were done in Part 1 in the 750‐ and 1500‐mg cohorts within 6‐h windows on day 1, then once daily on days 2 and 3 up to 72 h post dose. MMV367 plasma and urine concentrations were quantified *vs* an internal standard using a validated method with high‐performance liquid chromatography (HPLC) and high‐resolution mass spectrometry (Swiss Bioquant). The lower limit of quantification was 10 ng/mL in plasma and 2 ng/mL in urine.

A participant was evaluable for PK analysis if they received all the required study drug doses, completed safety and PK assessments up to 96 h post dose, and had no major protocol deviations or adverse events suggesting that the whole dose was not available for absorption. Plasma and urine PK parameters were estimated from concentration‐time profiles using non‐compartmental analysis (Phoenix WinNonlin version 8.3; Certara USA Inc.). Plasma PK parameters included maximum observed concentration (*C*_max_), time to maximum observed concentration (*T*_max_), area under the curve (AUC) from time 0 until 24, 48 and 72 h post dose (AUC_0‐24_, AUC_0‐48_, AUC_0‐72_), AUC until the last measurable concentration (AUC_0‐last_), AUC extrapolated to infinity (AUC_0‐∞_), percentage of AUC extrapolated from the time of the last measurable concentration to infinity (%AUC_extrap_), the apparent terminal half‐life (*t*_½_), apparent total clearance (CL/F), apparent volume of distribution (Vz/F), and renal clearance (CLr). The amount and percentage of drug excreted in urine were estimated. In Part 2, relative bioavailability was calculated using *C*_max_, AUC_0‐last_ and AUC_0‐∞_ as ([fed/fasted] × 100). In Part 3, the AUC for the defined interval between doses (AUC_0‐tau_) was calculated for days 1 and 3, and the exposure ratio was calculated for *C*_max_ and AUC_0‐tau_ for the single dose (day 1) *vs* the repeated dose (day 3).

### 2.8 Metabolite identification

Metabolite screening and identification were performed in human plasma and urine samples from the six participants who received a single dose of MMV367 1500 mg using HPLC and high‐resolution mass spectrometry by comparing the extracted ion chromatograms of exact masses of common modifications from full‐scan data for pooled predose control samples and postdose samples.

### 2.9 Statistical analysis

Analyses were conducted using SAS (version 9.4). Summary statistics are presented for safety and tolerability outcomes. Geometric mean, geometric standard deviation (SD) and geometric co‐efficient of variability (CV%) were presented for all plasma PK parameters except *T*_max_ (median, range). To evaluate dose proportionality, a formal statistical analysis was done for *C*_max_, AUC_0‐last_ and AUC_0‐∞_ across five single doses in the fasted state (100, 300, 440, 750 and 1500 mg). Using the power model, the slope of the log‐transformed dose was estimated as the independent variable in a linear regression. The log‐transformed PK parameter served as the response or dependent variable. Subsequently, the 90% confidence interval (CI) of the slopes was calculated. The power model was used to estimate the increase in the PK parameter resulting from a doubling of the dose and 90% CIs calculated.

For Part 2, the effect of a high‐fat meal on *C*_max_, AUC_0‐last_ and AUC_0‐∞_ was analysed for participants who completed both fed and fasted periods using mixed‐effect modelling, including terms for fed/fasted status and period as fixed effects and participant as a random effect using SAS PROC MIXED. The adjusted means, including differences from the fed/fasted comparison and the associated 90% CIs obtained from the model, were back transformed on the log scale to obtain adjusted geometric mean ratios and 90% CIs.

### 2.10 Nomenclature of targets and ligands

Key protein targets and ligands in this article are hyperlinked to corresponding entries in http://www.guidetopharmacology.org, the common portal for data from the IUPHAR/BPS Guide to PHARMACOLOGY,^10^ and are permanently archived in the Concise Guide to PHARMACOLOGY 2021/22.^4^

## 3 RESULTS

### 3.1 Participants

**FIGURE 1:**
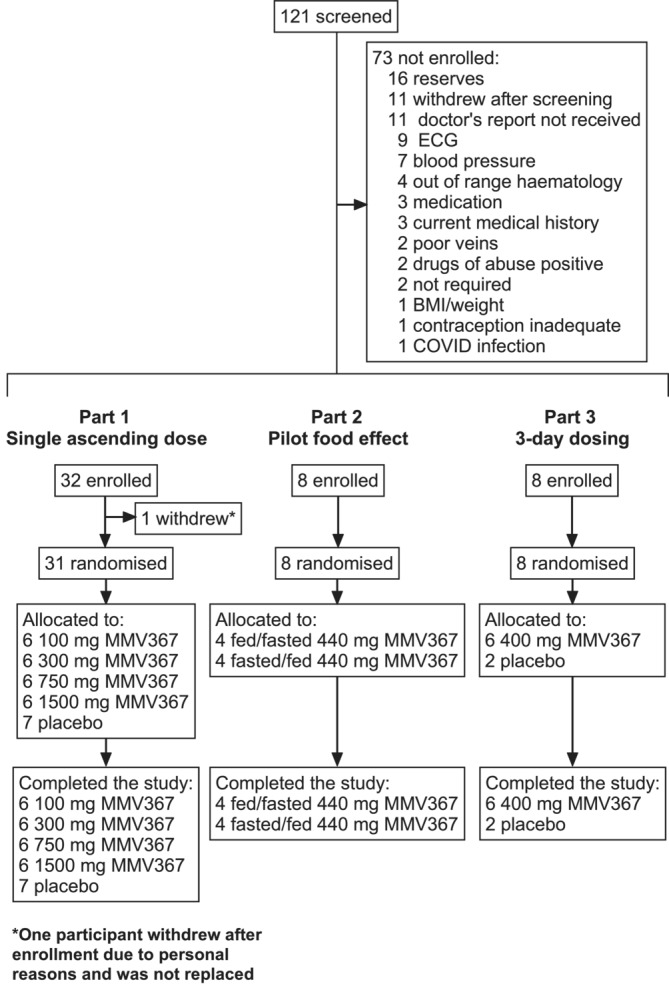
Participant disposition.

Forty‐seven participants were enrolled, with 38 receiving MMV367 and nine placebo (Figure 1). Baseline characteristics are shown in Table 1. Of the 11 females who received MMV367 in the study, eight were of childbearing potential: seven were using combined hormonal contraception and one abstinence. All participants completed the study, with no major protocol deviation, and were evaluated for safety outcomes and PK (Figure 1).

**TABLE 1:** Summary of demographic characteristics for participants who received MMV367 (100‐1500 mg) or placebo, safety analysis set.

Characteristic	Part 1					Part 2		Part 3	
	Placebo (n = 7)	100 mg (n = 6)	300 mg (n = 6)	750 mg (n = 6)	1500 mg (n = 6)	440 mg fed/fasted (n = 4)	440 mg fasted/fed (n = 4)	Placebo (n = 2)	400 mg (n = 6)
Mean age (SD) [range], years	40.1 (10.6) [27‐55]	44.0 (10.0) [29‐54]	35.2 (6.6) [25‐43]	41.3 (10.7) [28‐53]	36.3 (6.4) [25‐42]	37.3 (8.9) [29‐49]	29.3 (6.2) [22‐37]	39.5 (3.5) [37‐42]	30.7 (11.4) [23‐53]
Sex, n (%)									
Male	2 (28.6)	3 (50.0)	5 (83.3)	4 (66.7)	5 (83.3)	4 (100)	3 (75.0)	1 (50.0)	3 (50.0)
Female	5 (71.4)	3 (50.0)	1 (16.7)	2 (33.3)	1 (16.7)	0	1 (25.0)	1 (50.0)	3 (50.0)
Race, n (%)									
Asian	0	1 (16.7)	1 (16.7)	0	1 (16.7)	0	0	0	0
Black/African American	1 (14.3)	0	0	0	0	0	0	0	1 (16.7)
White	6 (85.7)	5 (83.3)	5 (83.3)	5 (83.3)	5 (83.3)	4 (100)	4 (100)	2 (100)	5 (83.3)
Other	0	0	0	1 (16.7)	0	0	0	0	0
Mean (SD) height [range], cm	169.4 (7.1) [161‐181]	167.8 (7.6) [154‐176]	174.8 (11.6) [160‐194]	171.7 (8.4) [162‐185]	175.3 (11.0) [158‐184]	184.8 (7.5) [176‐192]	174.8 (11.7) [159‐185]	178.5 (7.8) [173‐184]	171.5 (5.5) [166‐182]
Mean weight (SD) [range], kg	76.3 (12.8) [54.5‐93.5]	74.4 (9.8) [63.1‐87.7]	74.8 (11.9) [65.8‐96.8]	75.9 (12.5) [65.7‐98.1]	86.8 (13.8) [69.3‐103.5]	82.0 (9.3) [72.5‐93.9]	80.4 (10.2) [68.3‐90.7]	73.8 (17.3) [61.5‐86.0]	79.3 (3.7) [73.5‐82.9]
Mean BMI (SD) [range], kg/m^2^	26.6 (4.2) [21.0‐31.1]	26.4 (2.7) [22.1‐29.3]	24.4 (2.1) [22.2‐27.8]	25.7 (2.7) [22.8‐29.0]	28.1 (2.1) [24.9‐30.6]	24.0 (2.0) [21.5‐25.7]	26.4 (3.1) [22.8‐30.0]	23.0 (3.5) [20.5‐25.4]	27.0 (1.7) [24.6‐28.6]

Abbreviations: SD, standard deviation; BMI, body mass index.

### 3.2 Safety and tolerability

Overall, 26 TEAEs occurred in 36.8% (14/38) of participants administered MMV367 and seven in 44.4% (4/9) receiving placebo (Table 2). Across the MMV367 doses, headache was the most common TEAE, with no apparent relationship to dose, and was also observed in the placebo group (Table 2). There were no serious adverse events or adverse events leading to study withdrawal. Following MMV367, TEAEs were mild, except for one moderate case of orthostatic hypotension (MMV367 400 mg); this 53‐year‐old female reported feeling lightheaded approximately 6 h post dose on day 1, lasting 2 min with moderate postural hypotension with a drop in blood pressure (systolic −21 and diastolic −26 mmHg) recorded, which was not considered IMP related. Only two IMP‐related TEAEs were reported (abdominal pain and upper abdominal pain), in the same participant (MMV367 750 mg), which resolved spontaneously. TEAEs were mild in the placebo group, except one participant with a tooth fracture and toothache of moderate severity, not IMP related.

**TABLE 2:** Frequency of treatment‐emergent adverse events (TEAEs), safety analysis set

TEAE	Part 1					Part 2		Part 3	
	Placebo (n = 7)	100 mg (n = 6)	300 mg (n = 6)	750 mg (n = 6)	1500 mg (n = 6)	440 mg fed (n = 8)	440 mg fasted (n = 8)	Placebo (n = 2)	400 mg (n = 6)
Any TEAE	2 (28.6) [5]	1 (16.7) [1]	3 (50.0) [4]	2 (33.3) [5]	1 (16.7) [1]	2 (25.0) [2]	1 (12.5) [1]	2 (100) [2]	4 (66.7) [12]
Dizziness	1 (14.3) [1]	0	1 (16.7) [1]	0	0	0	0	0	2 (33.3) [2]
Headache	0	0	0	1 (16.7) [1]	0	1 (12.5) [1]	1 (12.5) [1]	1 (50.0) [1]	1 (16.7) [1]
Presyncope	0	0	1 (16.7) [1]	0	0	0	0	1 (50.0) [1]	1 (16.7) [1]
Abdominal pain	0	0	0	1 (16.7) [1]^a^	0	0	0	0	0
Abdominal pain upper	0	0	0	1 (16.7) [1]^a^	0	0	0	0	0
Diarrhoea	0	0	0	1 (16.7) [1]	0	0	0	0	0
Flatulence	1 (14.3) [1]	0	0	0	0	0	0	0	0
Toothache	1 (14.3) [1]	0	0	0	0	0	0	0	0
Dental paraesthesia	0	0	0	0	0	0	0	0	1 (16.7) [1]
Conjunctivitis	0	0	0	1 (16.7) [1]	0	0	0	0	0
Nasopharyngitis	0	0	0	0	1 (16.7) [1]	1 (12.5) [1]	0	0	1 (16.7) [1]
Rhinitis	0	0	0	0	0	0	0	0	1 (16.7) [1]
Influenza‐like illness	0	0	0	0	0	0	0	0	1 (16.7) [1]
Productive cough	0	0	0	0	0	0	0	0	1 (16.7) [1]
Backpain	0	0	0	0	0	0	0	0	2 (33.3) [2]
Tooth fracture	1 (14.3) [1]	0	0	0	0	0	0	0	0
Dysmenorrhoea	0	0	1 (16.7) [1]	0	0	0	0	0	0
Respiratory symptom	0	0	1 (16.7) [1]	0	0	0	0	0	0
Tooth extraction	1 (14.3) [1]	0	0	0	0	0	0	0	0
Orthostatic hypotension	0	1 (16.7) [1]	0	0	0	0	0	0	1 (16.7) [1]

*Note*: Data are number of participants (percentage of participants) [number of events].

^a^ IMP‐related TEAEs, both occurring in the same participant.

**FIGURE 2:**
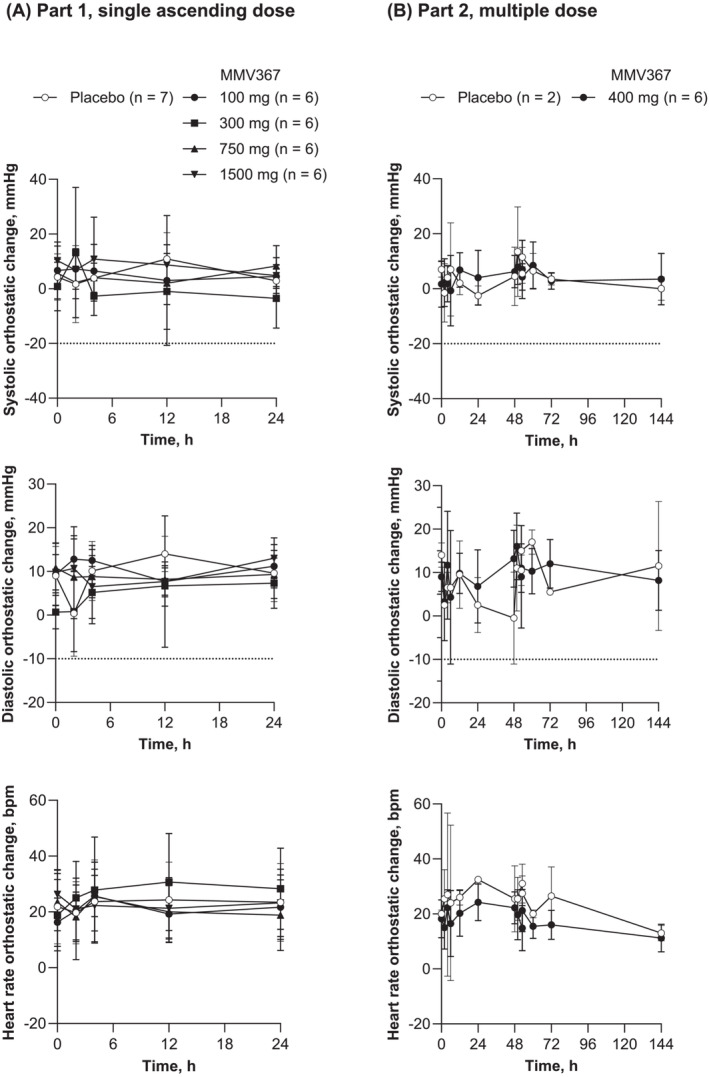
Orthostatic systolic and diastolic pressure and heart rate. Values are arithmetic mean ± standard deviation. Safety analysis set. (A) Part 1, MMV367 single ascending doses. Time 0 h represents the predose assessment. (B) Part 2, MMV367 doses on days 1, 2 and 3. Values at 0, 24 and 48 h were predose assessments. A notable decrease was defined as >20 mmHg systolic blood pressure and >10 mmHg diastolic blood pressure, shown by dotted lines.

There were no clinically important changes in haematology, coagulation, clinical chemistry or urinalysis findings from baseline with any MMV367 regimen or placebo. Neither were there any clinically relevant changes in vital signs. There was no trend for a drug‐induced effect on orthostatic blood pressure or heart rate (Figure 2).

**FIGURE 3:**
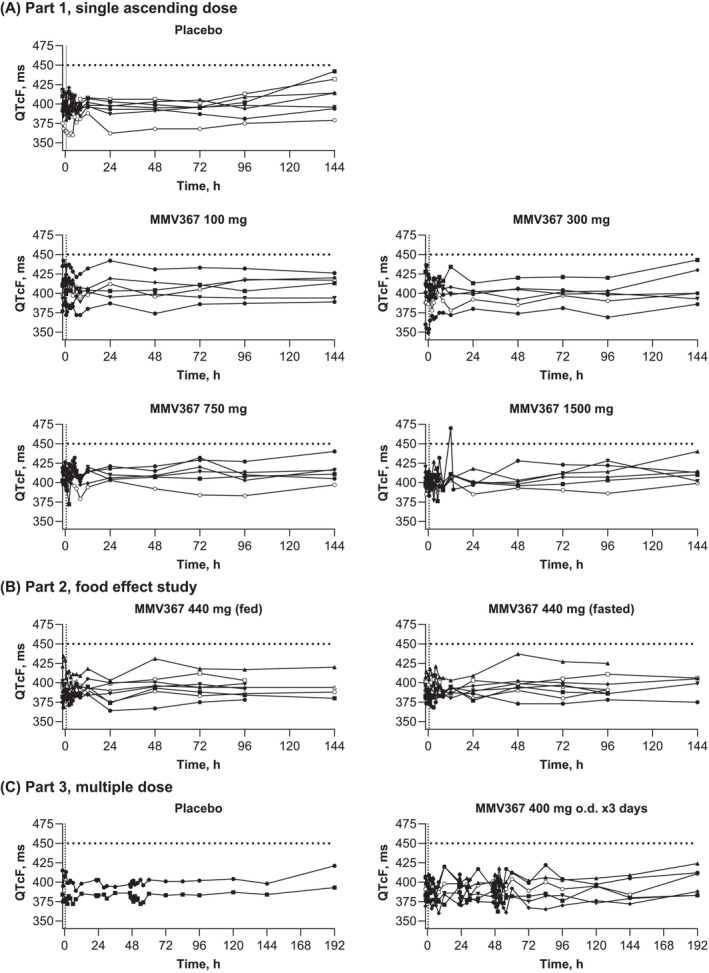
Individual participant Fredericia‐corrected QT interval (QTcF) values. Safety analysis set. Predose assessments are shown from time −1.5 to time 0 as shown by the vertical dotted line. The horizontal dotted line indicates increases >450 ms. For triplicate ECGs, the mean QTcF value is shown (48, 72, 96 and 144 h in Parts 1 and 2, and 47.5, 47, 48, 52, 54, 60, 84, 96, 120, 144 and 192 h in Part 3).

There were two Fridericia‐corrected QTc (QTcF) values outside the normal range: 470 ms at 12 h post dose, 64 ms increase from baseline (MMV367 1500 mg), an unscheduled ECG performed 1.5 h later showed 391 ms, and 492 ms at 144 h post dose (placebo) for the first of triplicate ECGs, with repeated values of 412 and 423 ms. There were no other QTcF values >450 ms or changes from baseline >60 ms across Parts 1, 2 or 3 (Figure 3). QTcF interval increases >30 to ≤60 ms were in Part 1, placebo (39 ms), MMV367 300 mg (37 ms), MMV367 1500 mg (40 ms), in Part 2, MMV 440 mg, fasted (34 ms), and not reported in Part 3. None of the ECG findings were clinically important or considered IMP related (Figure 3).

For taste/palatability, MMV367 dispersible granules administered as a suspension in water had a lower median overall liking score (4.00, dislike slightly) *vs* placebo (5.00, neither like nor dislike). Smell and sweetness had the highest median score (5.00) for MMV367 and grittiness the lowest (3.00, dislike moderately). No taste‐masking agents were used in the MMV367 or placebo formulations.

### 3.3 Single‐dose pharmacokinetics

**FIGURE 4:**
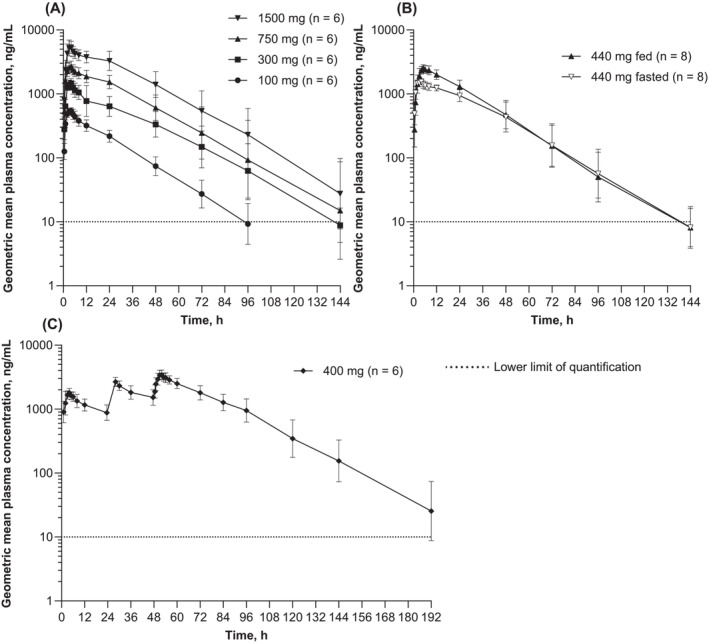
Geometric mean plasma concentration‐time curves for MMV367. (A) Part 1, single oral ascending doses. (B) Part 2, single oral dose in fed and fasted states. (C) Part 3, multiple oral doses on days 1, 2 and 3. Data are geometric mean ± geometric standard deviation. The horizontal dotted line represents the lower limit of quantification. Pharmacokinetic analysis set.

Geometric mean plasma concentration‐time profiles following MMV367 single oral doses (100, 300, 750 or 1500 mg) administered in the fasted state are shown in Figure 4A and PK parameters in Table 3. *T*_max_ ranged between 2.0 and 5.0 h post dose, with the geometric mean *t*_1/2_ ranging between 16.5 and 18.4 h. Geometric mean *C*_max_ values increased from 565 ng/mL at 100 mg of MMV367 to 5430 ng/mL at 1500 mg. Geometric mean AUC_0‐∞_ ranged between 13 200 and 184 000 ng·h/mL, and the between‐subject variability was low to moderate (<41.0%). Geometric mean Vz/F ranged between 179 and 217 L, and CL/F between 116 and 136 mL/min across the 100‐1500‐mg dose range. Urine PK was evaluated at the 750 and 1500 mg dose levels, with approximately 0.3% and 0.4%, respectively, of the administered MMV367 dose excreted unchanged in the urine, resulting in CLr values of 0.46 and 0.51 mL/min, respectively.

**TABLE 3:** MMV367 pharmacokinetics following a single ascending dose (Part 1, fasted), in the fed and fasted state (Part 2), pharmacokinetic analysis set.

Parameter	Part 1				Part 2	
	100 mg (n = 6)	300 mg (n = 6)	750 mg (n = 6)	1500 mg (n = 6)	440 mg fasted (n = 8)	440 mg fed (n = 8)
*T*_max_ (h)	4.0 (2.0‐4.0)	4.0 (4.0‐5.0)	4.0 (3.0‐4.0)	4.0 (3.0‐4.0)	3.5 (2.0‐24.0)	5.0 (4.0‐6.0)
*C*_max_ (ng/mL)	565 (9.3)	1480 (17.5)	2710 (19.1)	5430 (24.5)	1620 (7.5)	2610 (11.2)
AUC_0‐24_ (ng·h/mL)	8010 (16.3)	21 200 (28.1)	45 300 (18.9)	90 400 (22.0)	28 500 (8.8)	43 400 (13.6)
AUC_0‐48_ (ng·h/mL)	11 200 (17.5)	32 600 (31.1)	69 700 (21.3)	144 000 (27.8)	44 800 (15.8)	63 600 (18.5)
AUC_0‐72_ (ng·h/mL)	12 400 (17.8)	38 500 (33.0)	80 500 (26.2)	167 000 (31.6)	52 000 (21.7)	71 000 (22.0)
AUC_0‐last_ (ng·h/mL)	12 800 (18.7)	42 500 (35.5)	89 200 (37.9)	182 000 (36.6)	55 700 (26.7)	72 400 (25.4)^a^
AUC_0‐∞_ (ng·h/mL)	13 200 (18.1)	43 200 (34.6)	91 700 (41.0)	184 000 (36.9)	56 500 (26.7)	75 100 (24.9)
AUC_extrap_ (%)	3.15 (54.4)	1.23 (87.0)	1.67 (122.8)	0.631 (144.8)	1.18 (76.4)	0.768 (101.4)
*t*_1/2_ (h)	16.5 (26.3)	17.9 (23.5)	18.4 (54.9)	18.1 (19.3)	17.0 (13.8)	15.9 (21.0)
λz (1/h)	0.042 (26.3)	0.039 (23.5)	0.038 (54.9)	0.038 (19.3)	0.041 (13.8)	0.044 (21.0)
CL/F (mL/min)	126 (18.1)	116 (34.6)	136 (41.0)	136 (36.9)	130 (26.7)	97.7 (24.9)
Vz/F (L)	180 (29.5)	179 (45.4)	217 (18.5)	213 (21.3)	191 (18.6)	134 (10.1)
CLr (mL/min)	NA	NA	0.456 (45.4)	0.51 (38.5)	NA	NA

*Note*: Data are geometric mean (% coefficient of variance), except *T*_max_, which is the median (range). NA, not applicable.

Abbreviations: AUC_0‐24_, AUC_0‐48_ and AUC_0‐72_, area under the curve (AUC) from time 0 until 24, 48 and 72 h post dose; AUC_0‐last_, AUC until the last measurable concentration; AUC_0‐∞_, AUC extrapolated to infinity; AUC_extrap_, percentage of AUC extrapolated from the time of the last measurable concentration to infinity; *C*_max_, maximum observed concentration; *T*_max_, time to maximum observed concentration; *t*_½_, apparent terminal half‐life; λz, apparent terminal elimination rate constant; CL/F, apparent total clearance; Vz/F, apparent volume of distribution; CLr, renal clearance.

^a^ n = 7.

### 3.4 Effect of food on PKs

Geometric mean concentration‐time profiles following a single oral MMV367 dose (440 mg) administered in the fed or fasted state are shown in Figure 4B and PK parameters in Table 3. In the fasted state, *T*_max_ ranged between 2.0 and 4.2 h, except for one participant who had a secondary peak (1480 ng/mL) in MMV367 plasma concentration at 24 h, with an initial peak occurring at 4 h post dose. In the fed *vs* fasted state, the geometric mean Vz/F and CL/F were both reduced, and *t*_1/2_ was similar. There was a slight delay (1.5 h) to *T*_max_ in the fed state *vs* fasting. Compared to the fasted state, a high‐fat meal increased MMV367 exposure, with a geometric mean relative bioavailability of 161.4% (90% CI 148.3, 175.6) for *C*_max_, 130.4% (122.2, 139.1; CV% 9.0) for AUC_0‐last_, and 132.9% (124.1, 142.3) for AUC_0‐∞_.

### 3.5 Dose proportionality

Statistical assessment of dose proportionality (fasted state) across Parts 1 and 2 (100‐1500 mg MMV367) were 0.82 (90% CI 0.76, 0.88) for *C*_max_, 0.97 (0.87, 1.07) for AUC_0‐last_ and 0.96 (0.86, 1.06) for AUC_0‐∞_, with the 90% CIs for all parameters lying within the critical range (0.74, 1.26), indicating approximate dose proportionality. Predicted multiples for doubling the MMV367 dose were *C*_max_ 1.76 (90% CI 1.69, 1.84), AUC_0‐last_ 1.95 (1.82, 2.10) and AUC_0‐∞_ 1.94 (1.81, 2.09).

### 3.6 Multiple‐dose pharmacokinetics

Concentration‐time profiles following three daily MMV367 doses (400 mg) in a fasting state are shown in Figure 4C and PK parameters in Table 4. Median *T*_max_ was 4.0 h post dose on day 1. Following three daily doses, concentrations declined in a monophasic or biphasic manner, with a geometric mean *t*_1/2_ of 19.7 h. Steady state was not reached, and the geometric mean (geometric CV%) day 1:3 exposure ratios were 1.88 (4.9%) for *C*_max_ and 2.13 (7.7%) for AUC_0‐tau_.

**TABLE 4:** MMV367 pharmacokinetics following 400 mg once daily for 3 days (Part 3, fasted), pharmacokinetic analysis set.

Parameter	400 mg (n = 6)	400 mg (n = 6)
	Day 1	Day 3
*T*_max_ (h)	4.00 (2.00‐5.00)	3.50 (3.00‐5.00)
*C*_max_ (ng/mL)	1870 (16.6)	3500 (15.8)
AUC_0‐tau_ (ng·h/mL)	28 300 (20.9)	60 100 (18.4)
AUC_0‐∞_ (ng·h/mL)	NA	118 000 (29.4)
*t*_1/2_ (h)	NA	19.7 (22.5)
CL/F_tau_ (mL/min)	NA	111 (18.4)
Vz/F_tau_ (L)	NA	189 (23.5)
Day 1:3 exposure ratio *C*_max_	NA	1.88 (4.9)
Day 1:3 exposure ratio AUC_0‐tau_	NA	2.13 (7.7)

*Note*: Data are geometric mean (% coefficient of variance), except *T*_max_, which is the median (range). NA, not applicable.

Abbreviations: AUC_0‐tau_, area under the curve (AUC) over the dosing interval; AUC_0‐last_, AUC until the last measurable concentration; AUC_0‐∞_, AUC extrapolated to infinity; *C*_max_, maximum observed concentration; *T*_max_, time to maximum observed concentration; *t*_½_, apparent terminal half‐life; CL/F_tau_, apparent total clearance over the dosing interval; Vz/F_tau_, apparent volume of distribution over the dosing interval.

### 3.7 Metabolite identification

In plasma samples, a glucuronide of MMV367 was the most abundant metabolite and multiple products of oxidised MMV367 were identified. In urine samples, a glucuronide was most abundant followed by mono‐oxidised or di‐oxidised MMV367 and a mono‐oxidation/glucuronide conjugation.

## 4 DISCUSSION

In this phase 1a first‐in‐human study, MMV367 at single doses up to 1500 and 400 mg once daily for 3 days was well tolerated, with no apparent effect of a high‐fat meal on safety assessments and no clinically relevant food‐effect on PK.

Antimalarial medicines are widely used in resource‐poor settings, where managing adverse events can be challenging. Safety and tolerability are therefore key considerations. No concerning safety signals, serious adverse events or withdrawals were observed for MMV367. Two IMP‐related TEAEs (abdominal pain and upper abdominal pain) were reported, but no other gastrointestinal TEAEs of any cause occurred, including at the highest dose tested (1500 mg). There were no clinically relevant effects of MMV367 on ECG parameters. The MMV367 safety and tolerability profile across the dose range tested therefore supports its suitability as an antimalarial drug.

At the highest MMV367 dose level (1500 mg), the geometric mean AUC_0‐∞_ was 184 000 ng·h/mL, approximately 2‐fold below the AUC cap proposed for this study (376 000 ng·h/mL) based on the NOAEL from preclinical toxicity studies in the most sensitive species (dog). This limit was based on the cumulative AUC over 0‐7 days from the GLP study, a method accepted by regulatory agencies as providing a suitable safety margin for antimalarials with a maximum treatment duration of 3 days. With a human single‐dose minimally effective dose of 440 mg predicted from preclinical PD data, the therapeutic window appears sufficient for an antimalarial, although confirmation will depend on parasite killing kinetics in humans.

Children are particularly at risk from malaria and antimalarials are typically dosed by body weight. As many children cannot swallow tablets and dose adjustment is challenging, child‐friendly formulations, such as dispersible granules, are essential. Palatability is key, as unpleasant taste or texture can lead to spitting out or vomiting, risking underdosing and treatment failure.^11^ MMV367 taste and palatability evaluated in adults using a standardised objective questionnaire were acceptable, and differences relative to placebo are unlikely to be clinically relevant. These findings inform the development of child‐friendly formulations, although acceptability must be evaluated in paediatric populations.

Approximately 10 other antimalarial candidates are currently in development.^12^ Antimalarials must be deployed as fixed‐dose combinations to deter drug resistance selection, and the selection of a suitable partner drug is critical. Partner drugs must have different mechanisms of action; however, as MMV367 has a novel mechanism of action,^3^ potential combinations are not limited in this regard. Compatibility ideally requires a similar duration of action to avoid exposing one component as monotherapy. The *t*_1/2_ provides an indication, with the MMV367 *t*_1/2_ of 16.5‐18.4 h similar to that of other antimalarial candidates such as SJ733 (17.4 h),^13^ P218 (19.6 h)^14^ and cipargamin (24 h).^15^ Longer *t*_1/2_ values were reported for ZY‐19489 (50‐97 h),^16^ DSM265 (86‐118 h)^17^ and MMV533 (103.8‐127.2 h).^18^ Another indicator is the geometric mean Vz/F, which for MMV367 ranged from 179 to 217 L in the fasted state. This is relatively low compared to candidates such as MMV533 (274‐506),^18^ OZ439 (1570 L)^19^ and P218 (350‐1588 L),^14^ but similar to cipargamin (124‐194 L).^15^ More specifically, post‐treatment antimalarial activity profiles should be matched.^20^ While preclinical studies provide initial estimates of the minimally effective dose, human volunteer infection studies are needed to refine PKPD models and design effective drug combinations and dosing regimens for testing in phase 2 and 3 trials.

While MMV367’s PK profile supports a single‐dose regimen, matching its duration of action with a partner antimalarial might require up to 3 days of therapy, the maximum acceptable duration as per the standard of care. Following MMV367 400 mg once daily for 3 days, *T*_max_ was similar on day 3 (3.5 h) compared to day 1 (4.0 h). Exposure based on *C*_max_ was 1.88‐fold greater and AUC_0‐tau_ was 2.13‐fold greater on day 3 compared to day 1, indicating moderate accumulation for this dosing regimen.^21^ As no safety impact would be anticipated, a 3‐day dosing regimen of MMV367 could be considered if necessary.

A lack of significant food‐effect is important for antimalarial drug feasibility and safety. In this pilot investigation, a high‐calorie, high‐fat meal caused no clinically relevant food‐effect on MMV367 PK, increasing mean exposure by 1.61‐fold for *C*_max_, 1.30‐fold for AUC_0‐last_ and 1.33‐fold for AUC_0‐∞_ *vs*. the fasted state. The increased exposure with a high‐fat meal did not increase safety or tolerability observations when compared to the fasted state. The high‐fat meal is an experimental “worse‐case” sceniario,^22^ and further work may consider the effect of meal types more typical of malaria endemic regions with the definitive formulation.

Following single doses in the fasted state, median *T*_max_ ranged from 3.5 to 4.0 h, indicating gradual oral absorption. Following a high‐fat meal, *T*_max_ was slightly delayed to 5.0 h, possibly due to delayed gastric emptying. At higher fasted doses, secondary peaks in plasma concentration‐time profiles were observed at 12 and 24 h post dose, potentially reflecting enterohepatic recirculation, whereby MMV367 and its metabolites are released in bile in anticipation/response to food intake near these time points. One participant (440 mg, fasted) had an atypical profile, with a secondary peak and *T*_max_ of 24.0 h post dose, compared to the group median value of 3.5 h. The AUC_0‐∞_ for this participant was relatively high (96 100 ng·h/mL) compared to the group geometric mean (56 500 ng·h/mL), although their fasted *t*_1/2_ (19.4 h) was consistent with the group geometric mean (17.0 h). No specific cause was identified, but it may fall within the normal range of inter‐individual variability. Additional data are needed to fully characterise the MMV367 population PK.

A limitation of this study is that no taste‐masking agents were used in the taste/palatability assessments at this early stage of formulation development, potentially compromising blinding. Also, although metabolites were identified in this study, these were not quantified for PK analysis.

MMV367 had safety, tolerability and PK profiles following single or once‐daily dosing over 3 days compatible with the requirements for an antimalarial drug. Further investigation in a malaria volunteer infection study is warranted to provide the necessary PD data to develop the most suitable fixed‐dose combinations for testing in malaria patients.

## AUTHOR CONTRIBUTIONS

A.K. and S.C. (sponsor medical director) were responsible for the overall study design, data interpretation and directing trial activities. S.C. was responsible for overseeing safety aspects of the study in collaboration with A.C. N.S. (principal investigator) and S.M. were responsible for acquisition of the data and contributed to study design and interpretation of data. B.B. and L.S. were responsible for aligning clinical trial design with overall project strategy and provided operational support. E.L. and T.D. were responsible for PK data interpretation. A.J. was responsible for overseeing safety aspects of the first‐in‐human study and verification of underlying data. A.K., N.S., D.G., A.J., R.S., A.C., R.A.G., S.M., E.L., T.D., F‐J.G., L.S., B.B. and S.C. contributed to the study design and to the analysis and interpretation of data. The trial sponsor (MMV) designed the study in collaboration with its co‐development partner (GSK) and with input from all authors. All authors contributed to the manuscript, provided critical review and approved the final version. All authors had access to the primary data, accept responsibility for the accuracy and completeness of the data, and were involved in the decision to submit for publication.

## CONFLICT OF INTEREST STATEMENT

A.K. was employed at MMV when the study was designed and conducted, and is a current employee of Novartis, Biomedical Research, Basel, Switzerland. The content of this paper is the responsibility of the individual authors and neither the study nor this publication is associated with Novartis, Biomedical Research. A.J. is the owner of AKJ Consulting, which received financial support from MMV in connection with this study. N.S., S.M., E.L. and T.D. are employees of Quotient Sciences, which received financial support from MMV to conduct the study. B.B. and S.C. are employees of MMV. L.S., R.S., A.C., R.A.G. and F‐J.G. are employees of GSK and may hold shares in the company. D.G. is the owner and director of Mangareva SRL, which received financial support from MMV to review and interpret the study results.

## Data Availability

De‐identified participant data are available on reasonable request and with completion of a signed data access agreement from (https://www.mmv.org/about-us/contact-us) referencing this publication. Data will be available for at least 5 years from publication of this study.

## References

[1] World Health Organization . World malaria report. Published 2024. Accessed 20 December, 2024. https://www.who.int/teams/global-malaria-programme/reports/world-malaria-report-2024

[2] Gallego‐Delgado J . Pathology of severe malaria. Pathogens. 2023;12:12. doi:10.3390/pathogens12121389 PMC10746059 38133274

[3] Bopp S , Pasaje CFA , Summers RL , et al. Potent acyl‐CoA synthetase 10 inhibitors kill *Plasmodium falciparum* by disrupting triglyceride formation. Nat Commun. 2023;14(1):1455. doi:10.1038/s41467-023-36921-2 36927839 PMC10020447

[4] Alexander SP , Fabbro D , Kelly E , et al. The concise guide to pharmacology 2021/22: enzymes. Br J Pharmacol. 2021;178(Suppl 1):S313‐S411. doi:10.1111/bph.15542 34529828

[5] U.S. Department of Health and Human Services Food and Drug Administration Center for Drug Evaluation and Research (CDER) . Estimating the maximum safe starting dose in initial clinical trials for therapeutics in adult healthy volunteers. 2005. Accessed 14 February, 2024. https://www.fda.gov/regulatory‐information/search‐fda‐guidance‐documents/estimating‐maximum‐safe‐starting‐dose‐initial‐clinical‐trials‐therapeutics‐adult‐healthy‐volunteers

[6] European Medicines Agency . Strategies to identify and mitigate risks for first‐in‐human and early clinical trials with investigational medicinal products – Scientific guideline. 2017. Accessed 14 February, 2024. https://www.ema.europa.eu/en/strategies‐identify‐and‐mitigate‐risks‐first‐human‐and‐early‐clinical‐trials‐investigational‐medicinal‐products‐scientific‐guideline#ema‐inpage‐item‐18704

[7] US Department of Health and Human Services Food and Drug Administration Center for Drug Evaluation and Research (CDER) . Guidance for industry: food‐effect bioavailability and fed bioequivalence studies. 2002. Accessed 14 February, 2024. https://www.fda.gov/regulatory‐information/search‐fda‐guidance‐documents/food‐effect‐bioavailability‐and‐fed‐bioequivalence‐studies

[8] Bowman CJ , Becourt‐Lhote N , Boulifard V , et al. Science‐based approach to harmonize contraception recommendations in clinical trials and pharmaceutical labels. Clin Pharmacol Ther. 2023;113(2):226‐245. doi:10.1002/cpt.2602 35388453 PMC10083981

[9] Zann V , McDermott J , Jacobs JW , et al. Palatability and physical properties of potassium‐binding resin RDX7675: comparison with sodium polystyrene sulfonate. Drug Des Devel Ther. 2017;11:2663‐2673. doi:10.2147/DDDT.S143461 PMC5593397 28919716

[10] Harding SD , Armstrong JF , Faccenda E , et al. The IUPHAR/BPS guide to PHARMACOLOGY in 2024. Nucleic Acids Res. 2024;52(D1):D1438‐D1449. doi:10.1093/nar/gkad944 37897341 PMC10767925

[11] Schluterman HM , Linardos CG , Drulia T , Marshall JD , Kearns GL . Evaluating palatability in young children: a mini‐review of relevant physiology and assessment techniques. Front Pediatr. 2024;12:1350662. doi:10.3389/fped.2024.1350662 38390280 PMC10881860

[12] MMV Medicines for Malaria Venture . MMV's pipeline of antimalarial drugs. 2024. https://www.mmv.org/mmv-pipeline-antimalarial-drugs. Accessed 11 September, 2024.

[13] Gaur AH , McCarthy JS , Panetta JC , et al. Safety, tolerability, pharmacokinetics, and antimalarial efficacy of a novel *Plasmodium falciparum* ATP4 inhibitor SJ733: a first‐in‐human and induced blood‐stage malaria phase 1a/b trial. Lancet Infect Dis. 2020;20(8):964‐975. doi:10.1016/S1473-3099(19)30611-5 32275867

[14] Chughlay MF , Rossignol E , Donini C , et al. First‐in‐human clinical trial to assess the safety, tolerability and pharmacokinetics of P218, a novel candidate for malaria chemoprotection. Br J Clin Pharmacol. 2020;86(6):1113‐1124. doi:10.1111/bcp.14219 31925817 PMC7256114

[15] Leong FJ , Li R , Jain JP , et al. A first‐in‐human randomized, double‐blind, placebo‐controlled, single‐ and multiple‐ascending oral dose study of novel antimalarial Spiroindolone KAE609 (Cipargamin) to assess its safety, tolerability, and pharmacokinetics in healthy adult volunteers. Antimicrob Agents Chemother. 2014;58(10):6209‐6214. doi:10.1128/AAC.03393-14 25114127 PMC4187895

[16] Barber BE , Fernandez M , Patel HB , et al. Safety, pharmacokinetics, and antimalarial activity of the novel triaminopyrimidine ZY‐19489: a first‐in‐human, randomised, placebo‐controlled, double‐blind, single ascending dose study, pilot food‐effect study, and volunteer infection study. Lancet Infect Dis. 2022;22(6):879‐890. doi:10.1016/S1473-3099(21)00679-4 35247321

[17] McCarthy JS , Lotharius J , Ruckle T , et al. Safety, tolerability, pharmacokinetics, and activity of the novel long‐acting antimalarial DSM265: a two‐part first‐in‐human phase 1a/1b randomised study. Lancet Infect Dis. 2017;17(6):626‐635. doi:10.1016/S1473-3099(17)30171-8 28363636 PMC5446412

[18] Bestgen B , Jones S , Thathy V , et al. Safety, tolerability, pharmacokinetics, and antimalarial activity of MMV533: a phase 1a first‐in‐human, randomised, ascending dose and food effect study, and a phase 1b *Plasmodium falciparum* volunteer infection study. Lancet Infect Dis. 2024. Online ahead of print. doi:10.1016/S1473-3099(2024)00664-00669 39708824

[19] Moehrle JJ , Duparc S , Siethoff C , et al. First‐in‐man safety and pharmacokinetics of synthetic ozonide OZ439 demonstrates an improved exposure profile relative to other peroxide antimalarials. Br J Clin Pharmacol. 2013;75(2):524‐537. doi:10.1111/j.1365-2125.2012.04368.x 22759078 PMC3558805

[20] Hastings IM , Hodel EM . Pharmacological considerations in the design of anti‐malarial drug combination therapies – is matching half‐lives enough? Malar J. 2014;13:62. doi:10.1186/1475-2875-13-62 24552440 PMC3975950

[21] Li L , Li X , Xu L , Sheng Y , Huang J , Zheng Q . Systematic evaluation of dose accumulation studies in clinical pharmacokinetics. Curr Drug Metab. 2013;14(5):605‐615. doi:10.2174/13892002113149990002 23701162

[22] US Department of Health and Human Services Food and Drug Administration Center for Drug Evaluation and Research (CDER) . Assessing the effects of food on drugs in INDs and NDAs — clinical pharmacology considerations guidance for industry. 2022. Accessed 25 March, 2024. https://www.fda.gov/media/121313/download

